# Comparing the incidence of SARS-CoV-2 across age groups considering sampling biases - use of testing data of autumn 2021 in Belgium

**DOI:** 10.1186/s13690-023-01072-9

**Published:** 2023-04-23

**Authors:** Adrien Lajot, Laura Cornelissen, Dieter Van Cauteren, Marjan Meurisse, Ruben Brondeel, Christine Dupont-Gillain

**Affiliations:** 1 Department of Epidemiology and public health, Sciensano, Brussels, Belgium; 2 Data Science Institute, I-BioStat, Hasselt University, Hasselt, Belgium; 3 Institute of Condensed Matter and Nanosciences, Faculty of Bioscience Engineering, UCLouvain, Louvain-la-Neuve, Belgium

**Keywords:** COVID-19, Real incidence, Observed incidence, Positivity ratio, Selection bias, Belgium

## Abstract

**Background:**

To design efficient mitigation measures against COVID-19, understanding the transmission dynamics between different age groups was crucial. The role of children in the pandemic has been intensely debated and involves both scientific and ethical questions. To design efficient age-targeted non-pharmaceutical interventions (NPI), a good view of the incidence of the different age groups was needed. However, using Belgian testing data to infer real incidence (RI) from observed incidence (OI) or positivity ratio (PR) was not trivial.

**Methods:**

Based on Belgian testing data collected during the Delta wave of Autumn 2021, we compared the use of different estimators of RI and analyzed their effect on comparisons between age groups.

**Results:**

We found that the RI estimator’s choice strongly influences the comparison between age groups.

**Conclusion:**

The widespread implementation of testing campaigns using representative population samples could help to avoid pitfalls related to the current testing strategy in Belgium and worldwide. This approach would also allow a better comparison of the data from different countries while reducing biases arising from the specificities of each surveillance system.

## Background

Since March 2020, COVID-19 has impacted the daily life of millions of people in Belgium and worldwide. Non-pharmaceutical interventions (NPIs, i.e., public health actions not related to vaccination or medication that aim to prevent or slow down the transmission of SARS-CoV-2 in the community) have been widely used to reduce the burden of this epidemic on our societies. However, even though efforts have been put into understanding which measures (e.g., physical distancing, working from home, isolation of COVID-19 cases, quarantine for high-risk contacts of COVID-19 cases, lockdowns, etc.) were the most effective [1], it remains partly unclear given the complexity of such studies, especially due to confounding factors and heterogeneity in terms of populations and their environment. Moreover, strict lockdown strategies were extremely disruptive economically and mentally, and the impact of NPIs could differ between age groups. Indeed, successive surveys of the Belgian adult population have shown a clear age gradient in the undesired effects of NPIs. Age groups younger than 65 years old experienced a stronger impact on mental health (anxiety, depression) and social health (dissatisfaction with social contacts) than citizens above 65 years old [2]. In the literature regarding age-related SARS-CoV-2 transmission, the question of the role played by children in the epidemic was a hot topic. In order to design efficient age-targeted NPIs, a good view of the incidence of the different age groups and the intergenerational transmission dynamics was needed.

The real incidence (RI) should ideally be known to study age-specific SARS-CoV-2 incidences and compare between age groups. The SARS-CoV-2 RI at a given time can be expressed as the proportion of a population newly infected by SARS-CoV-2 within a defined time window. It was not feasible to test every individual in a population at any time. Thus, testing was only performed on a limited population sample within each time window. Therefore, RI was unknown, and we had to rely on proxies. However, the quest for the best proxy was hampered by sampling biases [3]. Indeed, testing served not only epidemiological but also clinical or infection prevention purposes. The testing strategy for SARS-CoV-2, designed to prioritise individuals for testing comprised tests (i) performed randomly on a representative sample of the population (*random*), (ii) performed for screening purposes in given situations (e.g. in collectivities, before hospital admission, before crossing a border, etc.) (*screening*), or (iii) targeted at individuals with a high likelihood of being infected (i.e. persons having symptoms – suspected COVID-19 case (SCC) or after high-risk contact (HRC)) (*targeted testing*). Depending on the testing strategy, the test data may therefore have suffered from a lack of representativeness of the general population, which may have differed between age groups.

**Fig. 1:**
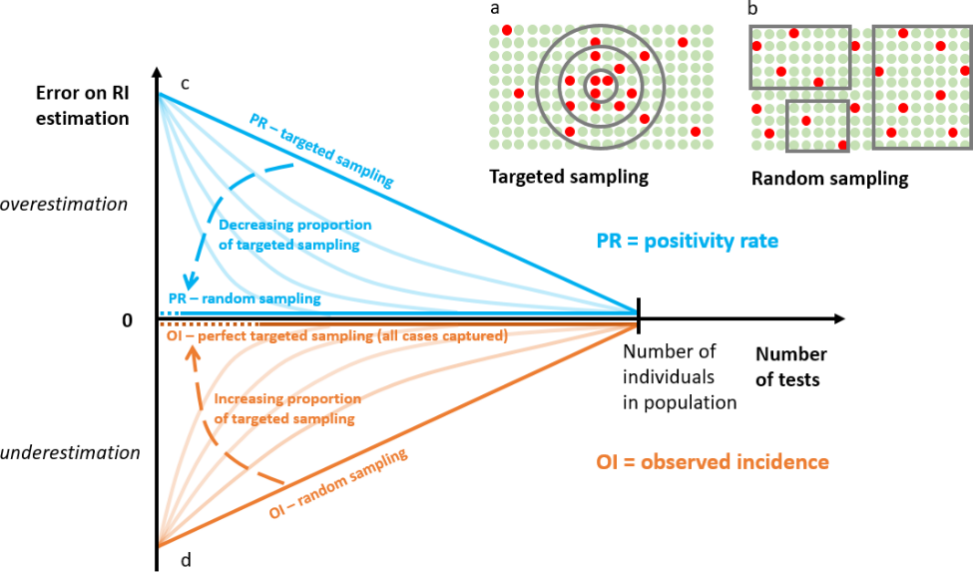
Number of captured cases as a function of the number of tests performed for a perfect targeted sampling strategy (a) and the case of a fully random strategy (b). Relation between the number of performed tests and the error on RI estimation based on (c) PR and (d) OI as a function of the testing strategy

The observed incidence (OI), i.e. the registered number of positive tests recorded in a given time window divided by the size of the population, has been largely used as a proxy of the RI. In Belgium, the public health Institute Sciensano publishes the number of positive tests in the last 14 days per 100,000 inhabitants [4]. As not all newly infected individuals are captured, the value of OI underestimates RI [5] (Fig. 1D). If more tests are performed, more cases are captured, and the value of OI gets closer to the one of RI (Fig. 1D). OI becomes equal to RI if the whole population is tested in the given time window (Fig. 1D). For a given number of performed tests, the underestimation decreases (i.e. OI becomes closer to RI) as the proportion of *targeted* sampling increases (Fig. 1D). Indeed, the probability of capturing a positive case when a small number of tests is performed is higher in targeted sampling than in random sampling (Fig. 1A,B). If the testing strategy for capturing cases tended to be perfectly efficient, OI would theoretically progressively reach RI while testing less than the whole population. It is impossible to capture all cases even with a rigorous tracing procedure in the real world.

The positivity rate PR (i.e. the number of positive tests divided by the total number of tests performed over a certain period) has been proposed as an additional indicator to account for differences in the intensity of testing [6, 7]. In contrast with OI, PR provides an overestimation of RI (Fig. 1C), especially if targeted testing is performed for which the probability of a positive test is higher than in the general population (Fig. 1C). For a given number of performed tests, the more the tested population is representative of the total population (moving from a *targeted* sampling to a *random* sampling), the more accurately PR estimates RI [5] (Fig. 1C).

When age groups must be compared to study their respective incidence and transmission dynamics, great care must be taken to avoid biases related to the choice of indicators and data selection. In many studies, OI was directly compared between age groups [8, 9]. However, this comparison implied that the RI underestimation degree, due to the use of OI, was comparable across age groups. This assumption was an important limitation of such studies, as the testing strategy may not have been uniform across the age groups. For example, some age groups may have been subject to more additional screening tests than others. Several studies also acknowledged age-dependent access to testing as a limitation of comparing OI between age groups [8–10]. OI is strongly dependent on the intensity of testing. If we could assume that SCCs are equally tested regardless of age; and if HRCs are equally traced and tested (regardless of age or the age of the index case). Then, OI could be directly compared between the different groups. Targeted testing data may be more accurate than screening data when OI is used as a proxy for RI.

**Fig. 2:**
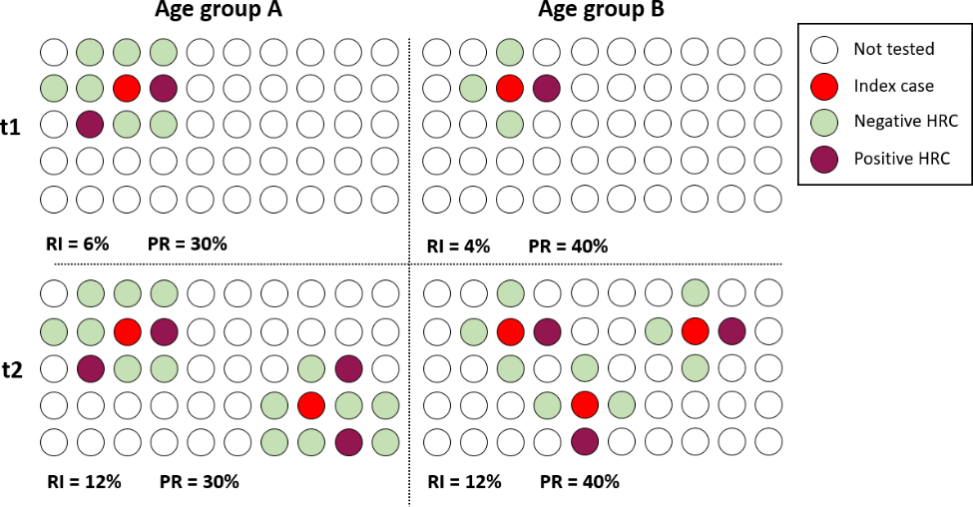
Potential bias when considering positivity rate. RI: Real Incidence, PR: Positivity rate. Age group A’s individuals make more contacts but are less infectious or susceptible than age group B’s individuals

In other studies, PR was used as a proxy for RI to limit the bias due to a heterogeneous number of tests across age groups [6, 7]. However, a direct comparison of PR implied that the indications for testing were similar across age groups, which is unlikely always to hold. Suppose the dataset included a mix of screening and targeted testing, in the proportion that varied according to age. In this case, some age groups may have featured a PR closer to the RI than others, leading to inaccurate comparisons and conclusions. As PR is a better estimator/proxy for random testing, data from screening tests (if no random sampling was put in place) may be more appropriate than data from targeted tests. Indeed, even if the representativeness of the tested populations would be considered similar, comparing PR of targeted tests between age groups could still lead to potential bias because, in this case, PR measures the probability of being positive when testing SCCs or HRCs. This probability can be the same between two age groups or at different time points even if the RI is different, as illustrated by the example depicted in Fig. 2. Let us consider two different age groups. Age group A has more HRCs (e.g., because they attend a collectivity such as a school) than age group B (e.g., they work at home and only have contact with their family members). However, age group A is less infectious or susceptible than age group B. In other words, HRC infection probability is lower for age group A than for age group B. At time t1, RI is higher for A (6%) than for B (4%); at time t2, RI is equal to 12% for A and B. At time t1, PR among targeted testing is higher for group B than for A, although the RI is higher for A than for B. At time t2, PR among targeted testing is higher for B than for A, although the RI is equal in both groups. Between t1 and t2, PR was constant for both groups (i.e. no growth), while RI growth had a factor of 2 for A and 3 for B.

Moreover, the intensity of the virus circulation can lead to a saturation of the contact tracing performance, with HRCs that are less reported and tested. In this situation, the PR among targeted tests measures the probability of being positive when having symptoms. Susceptibility to other pathogens leading to the same symptoms of SARS-CoV-2, which can be age-dependent, can modify this probability, leading to an additional bias. This bias also plays a role when considering a mix of HRCs and SCCs.

To sum up, the degree of bias of RI depends on the testing strategy and therefore the degree of bias could be age-dependent. The availability of additional information at the age group level (i.e. number of tests, number of targeted tests, number of screening tests, etc.) allows for a better comparison of RI between age groups. For example, age groups could be better compared using PR or OI calculated among screening or targeted tests, respectively.

### Methods

#### Data

SARS-CoV-2 testing data were collected through the national COVID-19 surveillance system implemented by Sciensano to monitor daily trends of virus circulation in the Belgian population [11]. Data on SARS-Cov-2 tests carried out between October 4, 2021, and December 26, 2021, were included in the study. These dates correspond to the official period used in Belgium to describe the wave driven by the Delta variant [12]. The sampling date and the infected person’s age were collected for each recorded positive test. These data were aggregated to obtain the number of confirmed positive tests by day and age group. Tests were performed using either PCR or antigen rapid detection test performed by a healthcare worker (with the latter accounting for only 15% of the data). The different testing indications (where “indication” stands for the reason for an individual to be submitted to a test) can be summarized as follow:

1. Suspected COVID-19 case (SCC), based on the presence of symptoms that fulfilled the case definition [13].
2. High-risk contact (HRC) with an individual with a recently confirmed positive SARS-CoV-2 test.
3. Screening before hospital admission for a non-Covid-19-related reason.
4. Screening for other reasons than before hospital admission (e.g. pre-holidays, access to an event, collectivities, etc.)

Indications 1 and 2 was designated as “targeted tests”. Population data were obtained through the Belgian statistical office, STATBEL, for the year 2021 [14]. All data analyses were conducted using R 4.0 [15].

#### Estimation methods of RI

In this work, we aimed to compare the estimation of the RI for each age group j, designated as $\widehat{{RI}_{j}}$, during the wave driven by the delta variant of SARS-CoV-2 in Belgium, based on the 4 following methods.

Method 1: OI considering all tests for an age group j, defined as the total number of positive tests in the last 14 days in age group j, multiplied by 100 and divided by the population size of age group j.

Method 2: OI considering only targeted tests for an age group j, defined as the total number of positive tests (in the last 14 days) for which the indication of testing was either SCC or HRC in age group j, multiplied by 100 and divided by the population size of age group j.

Method 3: PR considering all tests for an age group j, defined as the total number of positive tests in the last 14 days in age group j, multiplied by 100 and divided by the total number of tests performed in the last 14 days in age group j.

Method 4: PR considering only screening tests before hospital admission, for an age group j, defined as the total number of positive tests in the last 14 days for which the indication of tests was screening before hospital admission in age group j, multiplied by 100 and divided by the total number of tests in the last 14 days for which the indication of tests was screening before hospital admission in age group j.

For methods 1 and 3, based on all tests, the used data include indications of testing 1 to 4 (see above) and tests with unknown indications. Note that methods 2 (OI on targeted tests) and 4 (PR on screening data that are closer to random sampling) are expected to produce less error on the estimation of RI than methods 1 and 4 for a given number of performed tests (Fig. 1) and were thus explored to decrease bias.

For methods 3 and 4, considering PR, 95% confidence intervals were calculated using a normal approximation. For methods 1 and 2, considering OI, no 95% confidence intervals were calculated.

To facilitate the comparison of the different estimating methods, $\widehat{{}_{}}$the relative real incidence estimator ($\widehat{{RRI}_{j}}$) was calculated for each jth age group using 65 + age group as reference (i.e. the age group with the lowest $\widehat{{RI}_{ }}$ according to all estimating methods).

## Results

**Fig. 3:**
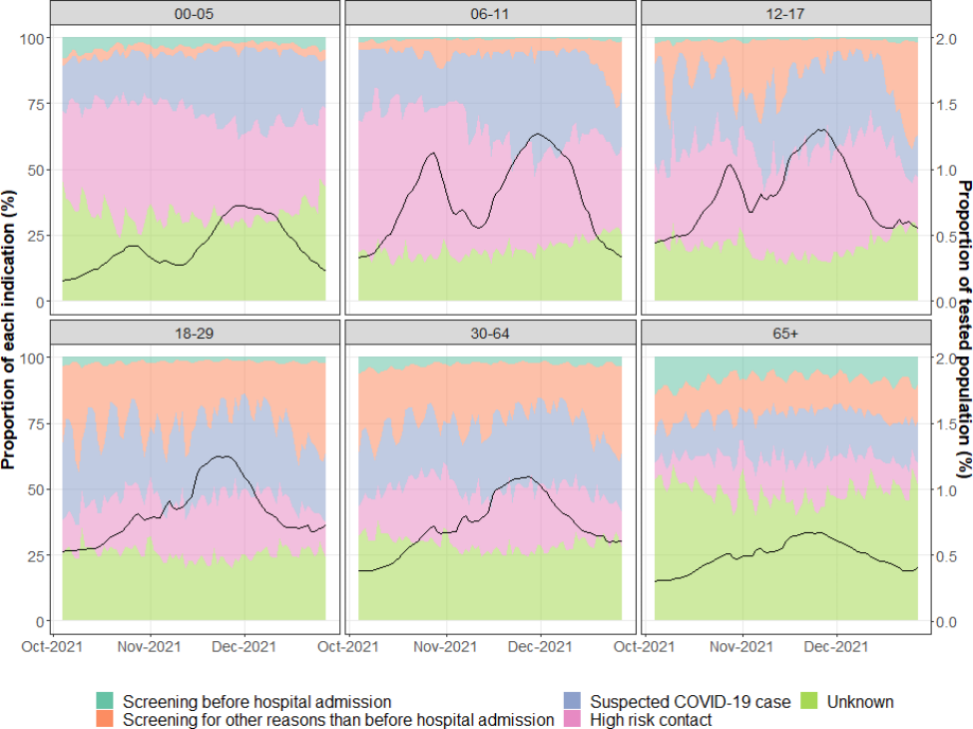
Evolution of the proportion of the tested population (black line) and evolution of the proportion of the different indications for testing over time (4 October 2021–26 December 2021) and by age group j (age 0–5, 6–11, 12–17, 18–29, 30–64, 65+) in Belgium

Figure 3 presents the proportion of the different indications for testing over time for each age group between October 4, 2021 and December 26, 2021, together with the proportion of each age group population that was tested. It reveals a varying fraction of screening vs. targeted testing by age group. The proportion of tests in a screening context was lower for younger children (aged 0 to 11 years old) than for adults. On the contrary, the proportion of targeted tests was higher for younger children, especially for 6–11 years old (about 75% of targeted tests), compared to people aged 65 + years old (about 25–30% of targeted tests). For 29% of the records, the indication of testing was unknown, and this proportion was highest for 65+ (≈ 50%) and lowest (≈ 20%) for children aged 6 to 17 years old.

Concerning the number of tests carried out according to age and time, Fig. 3 shows that the fraction of the tested population, which ranged from about 0.2 to 1.3%, was, in general, higher for 6–29 years old and lower for 0–5 children and for 65 + years old. Fewer tests were performed at the onset and the wave end, and more tests were performed at the heart of the wave. The temporary drop in early November in 0–17 years old may be attributed to a school holiday.

**Fig. 4:**
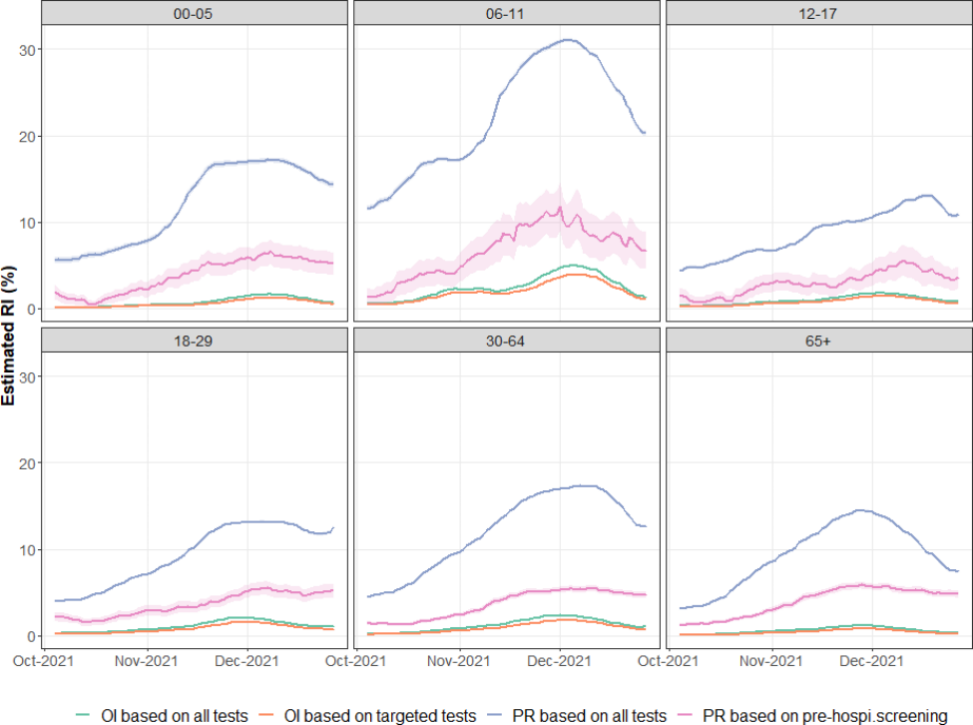
Evolution of $\widehat{{\text{R}\text{I}}_{\text{j}}}$ (%) over time (4 October 2021–26 December 2021) and by age group j (age 0–5, 6–11, 12–17, 18–29, 30–64, 65+) in Belgium, using OI considering all tests (green line), OI considering only targeted tests (orange line), PR considering all tests (blue line), and PR considering only screening tests before hospital admission (pink line)

Figure 4 shows, for each age group j between October 4, 2021, and December 26, 2021, the estimated RI, designated as $\widehat{{RI}_{j}}$, for the four estimation methods. Note that the 95% confidence interval was broader for PR on pre-hospitalization screening due to the smaller sample size (particularly in younger age groups). It can be seen that $\widehat{{RI}_{j}}$, was very sensitive to the estimation method. First, as expected (Fig. 1), for all the age groups, $\widehat{{RI}_{j}}$ estimated from OI was consistently lower than $\widehat{{RI}_{j}}$ estimated from PR. Second, $\widehat{{RI}_{j}}$based on PR considering all tests (Fig. 4) was higher than $\widehat{{RI}_{j}}$ based on PR considering only screening tests before hospital admission (Fig. 4) for all age groups. Third, $\widehat{{RI}_{j}}$ based on OI of all tests was higher than $\widehat{{RI}_{j}}$ based on OI of only targeted tests for all the age groups, even though the difference was not substantial. Moreover, for all the estimation methods, a higher $\widehat{{RI}_{j}}$ was observed among children aged 6 to 11 years old than for other age groups. Finally, all methods allowed to follow-up increasing or decreasing trends of the epidemic and showed a wave pattern to some extent. However, the speed of increase and absolute levels differed across methods.

**Fig. 5:**
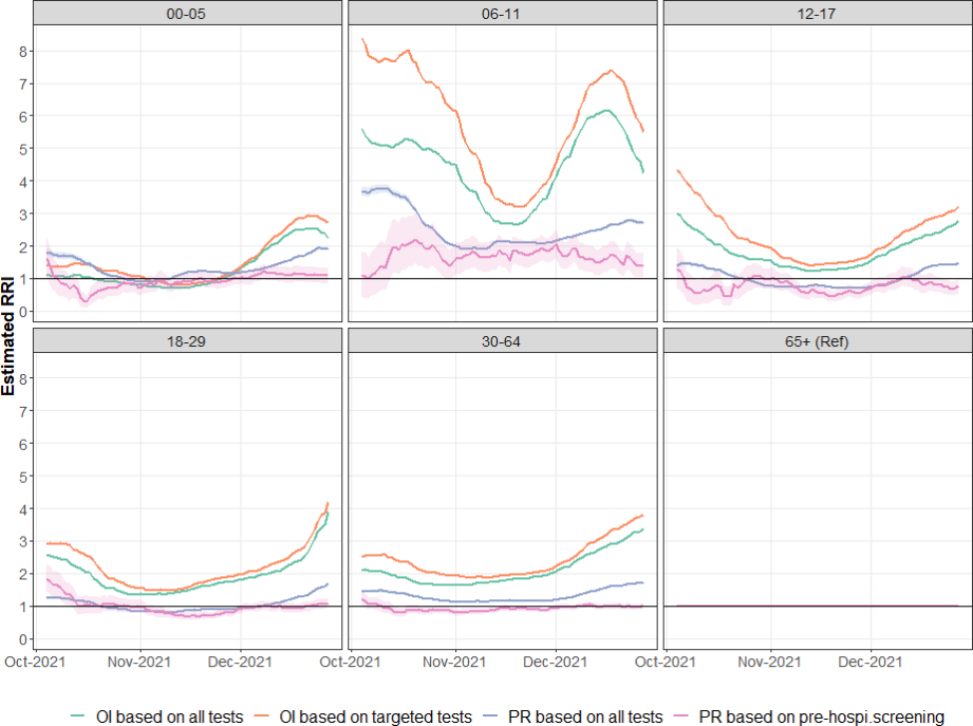
Evolution of $\widehat{{\text{R}\text{R}\text{I}}_{\text{j}}}$ over time (4 October 2021–26 December 2021) and by age group j (age 0–5, 6–11, 12–17, 18–29, 30–64, 65+) in Belgium, based on OI considering all tests (green line), OI considering only targeted tests (orange line), PR considering all tests (blue line), and PR considering only screening tests before hospital admission (pink line)

Figure 5 presents the estimated relative RI calculated using RI of 65 + years old as a reference, designated as $\widehat{{RRI}_{j}}$, for each age group j between October 4, 2021 and December 26, 2021 and by estimation method. It can be seen that $\widehat{{RRI}_{j}}$was also sensitive to the estimation method. First, the differences between age groups were most pronounced when using OI based on targeted testing. This aspect was especially true for the wave’s beginning and end and was most visible in the 6–11 age group. When considering OI based on all tests, large differences between age groups continued to exist but were somewhat dampened, especially for the 6–11 age group. Differences between age groups decreased further when considering PR, considering all tests or only hospitalization screening. For the latter, the ratio between different age groups was remarkably stable and close to 1 (meaning no difference in PR between the different age groups). Only for children aged 6–11, PR based on hospitalization screening remained somewhat higher than those of other age groups. Children aged 6 to 11 years old had the highest $\widehat{{RRI}_{j}}$ regardless of the estimating methods. Finally, it can be noted that all ${RRI}_{j}$values were lower at the heart of the wave around mid-November 2021, while they mostly featured higher values at the onset and the end of the wave.

## Discussion

A testing strategy needs to reconcile different objectives and constraints. The main objectives include infection prevention, contact tracing, epidemiological follow-up and clinical decision-making. Constraints can be logistical (access to testing, timeliness of results, sampling resources) and financial. For this reason, the testing strategy during the delta wave of COVID-19 in Belgium and most countries was mainly based on targeted sampling, which can cause a biased view of the real incidence [3, 16]. Moreover, as the fraction of screening vs. targeted testing varied according to age, comparisons between age groups are impeded and must thus be carried out with great caution. Given that testing strategies changed from one country or even region to another, comparing incidence and its growth by age group between countries is also subject to caution. Sound exploitation of testing data can thus only be delivered based on a deep knowledge of the testing strategy.

In this work, we explored the use of different estimators of RI intending to decrease bias. On the one hand, RI may be approached better by selecting the best subset of data according to the testing strategy. In this perspective, given the limited fraction of the population that can be tested, using PR on screening tests performed before hospital admission is expected to cause a lower bias on the estimation of RI. Indeed, the true RI value must be above OI measured on all tests. Since only a small fraction of the population was tested and, despite targeting, not all individuals that would have tested positive could be captured (see more details hereunder). It is also clear that targeted tests’ PR must be well above RI (Fig. 1). Using the data available in Belgium, PR on screening performed upon hospital admission may be the best choice to estimate RI (more details hereunder). On the other hand, when comparing age groups, a bias in the estimation of RI may be acceptable as long as this bias remains identical for all age groups. Indeed, the estimator could then be compared between age groups, delivering a correct view of the dynamics of the epidemic from one age group to another despite being biased.

To eliminate the effect of a variable fraction of screening vs. targeted tests according to age, taking into account only positive tests from targeted testing could be a good starting point. Compared to the classical method in which all tests are considered, the results obtained with this estimator showed more marked differences between age groups (Figs. 4 and 5). Observed differences were especially pronounced for children age groups. Children were less subject to screening (Fig. 2). However, this method assumes that the probability of an infected person being captured by the testing-tracing system is the same across all age groups. This hypothesis is only partially acceptable. Previous analyses of the contact tracing system in Belgium have shown that only 49% of index cases reported any HRCs. The average number of reported HRCs was 2.7 and did not increase when fewer physical distancing measures were in place. This situation probably indicated some degree of underreporting of HRCs through the central contact tracing center [17]. As in collectivities, local contact tracing took place. It is likely that the probability of finding HRCs from age groups attending collectivities, such as children at school, was higher than for other age groups. This imbalance then could caused an overestimation of the incidence in young age groups compared to older ones. This argument was frequently used by the educational sector and the Belgian pediatric Covid-19 Task Force in the public debate [18]. However, contact matrices also showed that some age groups, especially children, made more contacts than others [19]. It would then follow that these age groups could have more HRCs than others, even if the incidence is equal (Fig. 2).

Another potential solution to eliminate the bias due to the variable fraction of screening vs. targeted tests according to age could be to only look at the PR of screening tests performed on the occasion of non-COVID-19-related hospital admission. This method assumes that the population undergoing a SARS-CoV-2 test before hospital admission is representative of the entire population. However, people in this sub-population may have more comorbidities and different susceptibility to infection than the general population and might therefore show different behavior. This hypothesis is, thus, again, only partially acceptable. The subpopulation admitted for trauma care (e.g. fractures) might be more representative of the general population than patients coming in, e.g. chemotherapy. The data available in Belgium did, however, not allow to make this distinction. Another limitation of the hospital screening data is the relatively low fraction of the population tested in this framework, leading to more uncertainty, especially for younger age groups. Heireman et al. [20] also used PR of screening tests performed on the occasion of non-COVID-19-related hospital admission in Belgium to assess the SARS-CoV-2 circulation among patients without COVID-19 symptoms during the autumn wave of 2020. Among other findings, they observed that the peak of PR considering all tests occurred one month before the peak of PR considering only pre-hospitalization screening tests (October instead of November 2020). They assumed this discrepancy might be attributed to a modification of the testing strategy during the wave. Indeed, because of the fast increase of contaminations and limited testing capacity, tests were restricted to symptomatic individuals starting from October 2020. This situation highlights that changes in testing strategy may profoundly affect the evolution of indicators, which may distort data when age groups are compared. Here, for the Delta wave, the peak occurred roughly simultaneously for all the estimation methods (Fig. 3). This occurrence can be linked to the testing strategy that remained more stable than in 2020, when contaminations increased at the end of 2021.

Treating data from selected subsets is only made possible by accurately reporting the indication for testing, which was unknown for 29% of the records (Fig. 3). There are reasons to believe that these were not missing at random, and indications of missings were more frequent in the older age groups. Using different software packages in hospitals could lead to those missing indications [21]. As tests with unknown indications were discarded when only a subset of data was used (OI on targeted tests; PR on prehospitalization screening), this can, unfortunately, introduce a new bias in estimation methods that are, in principle, used to reduce sampling bias.

The above considerations were centered on using “real life” testing data acquired in Belgium to compare them between age groups even though the estimation of RI is biased, not focusing on the accuracy of the estimated incidence itself. Establishing the absolute incidence value for each age group may be more powerful. During the Delta wave, on the whole, children aged 6 to 11 years old seemed to experience a higher incidence than other age groups (Fig. 4). A seroprevalence study conducted in Belgium in primary schools confirmed the high number of new infections during the Delta wave [22]. Whereas the seroprevalence was estimated at 26.6 (21.5–32.8 ) % between September 20, 2021 and October 8, 2021, it quickly rose to 50.9 (43.7–59.2) % between December 06, 2021 and December 17, 2021.

Moreover, there is evidence that the OI from all tests strongly underestimated the RI in this age group: only 26.9% (so half of those with detectable SARS-CoV-2 antibodies) in this study reported a previous confirmed positive PCR or rapid antigen test. This latter figure is comparable to the cumulative percentage of people in that entire age group having tested positive according to testing data in mid-December 2021 (24%). These findings suggest that only half of the positive SARS-CoV-2-carrying primary school pupils were detected through the surveillance system. It was expected that OI would provide an underestimation of RI (Fig. 1), but it may well be that the extent of underestimation varied from one age group to another. Unfortunately, the high level of vaccination in adults prevented from inferring the proportion of cases captured by the surveillance system from seroprevalence data for older age groups (as antibodies are no longer only a marker of infection). As demonstrated by our results, differences in estimated incidence were sensitive to the method used for estimation (Fig. 5). Therefore, it is hard to know to which extent the RI in children was higher than in adults. However, differences in estimated incidence based on the PR considering only pre-hospitalization tests, which is likely the least biased, tended to be lower than the one based on the other methods (Fig. 5). This difference indicates that methods based on OI and PR considering all tests could overestimate differences in RI and, therefore, not adequate for comparisons between age groups.

Measuring RI is better achieved by designing a specific testing strategy. A prevalence study from England (REACT-1) [23] involving cross-sectional surveys of viral detection (virological swab for RT-PCR) tests in repeated samples of 100,000 to 150,000 randomly selected individuals across England showed the highest prevalence for 5–12 and 13–17 years old during the Delta wave. Riley et al. [24] compared the odds ratio of infection for each age group (compared to 35–44 years old) estimated from REACT-1 data [25] and the UK routine surveillance system data [26] during the first and second wave of COVID-19 in England. They found that, at that time, data targeting symptomatic cases from the routine surveillance system underestimated the odds ratio for children concerning REACT-1 data. Riley et al. [24] concluded that studies based on random sampling, such as REACT-1, that allow an accurate estimation of virus circulation, were a cornerstone of the design of adequate epidemic management policies.

Finally, it is worth mentioning that a high incidence in an age group at a given time is not sufficient to conclude that this age group is the main driver of an epidemic. In a study related to Influenza, Worby et al. [27] argued that an age group that is driving the epidemic would have a higher incidence before rather than after the peak of contamination observed for the whole population (i.e. for all age groups together). Therefore, the age group with the highest incidence at the end of an epidemic wave is not likely to be the driver of that epidemic wave. To better understand transmissions between age groups, a model taking into account the difference in estimated incidence and its time-dependent evolution, age-specific susceptibility, infectiveness, and contact rates between age groups would be particularly useful.

Based on these results and discussions, it must be highlighted that studying the role of different age groups in a pandemic such as the COVID-19 one is critical for designing efficient mitigation strategies and made difficult by sampling biases. When trying to exploit “as acquired” data from national surveillance systems, it is recommended to use subsets of data that compare age groups better, even if the true value of the incidence is not known. This approach requires the knowledge of indications for testing. Without a random sampling strategy, great care should be taken to record these indications uniformly across different regions and age groups. In this era where testing is accomplished at a large scale and data is made publicly available worldwide, it is crucial to raise awareness of biases that can impede comparisons between age groups and between countries or subpopulations.

Running a surveillance system based on random sampling is highly recommended from the start of an epidemic. In parallel with a more targeted approach, aiming at isolating contaminated individuals, this approach allows a much more robust epidemiological follow-up, with positive consequences on public health decisions.

## Conclusion

Deep knowledge of the role of different age groups in the dynamic of the epidemic was needed to tailor well-targeted efficient mitigation strategies. Due to the selection bias inherent to the surveillance system in Belgium, as in most other countries, this was, however, not trivial since these biases impeded comparisons between age groups. Here, we show that using well-selected subsets of data, delimited based on testing indications, reduces the bias occurring when comparing age groups. Hence, the positivity rate of screening tests performed upon hospital admission showed little differences between age groups during the Delta wave in Belgium, even though a slightly higher incidence seems to prevail in the 6–11 age group. This result is still imperfect, notably due to unknown testing indications in about one-third of the tests. This fact highlights the need to accurately record testing indications in regular surveillance systems, to increase the quality of further epidemiological analyses.

The widespread implementation of a repeated cross-sectional survey involving the collection of virological swabs from a series of age-stratified representative population samples, such as those performed in the UK [25], could help to avoid pitfalls related to the current testing strategy in Belgium and worldwide. This representativeness would also, beyond comparisons among age groups, open the door to using larger sets of data from different regions, ethnical groups, family settings, etc., while reducing biases arising from the specificities of each surveillance system.

## Data Availability

Data concerning the total number of positive tests by age group and the total number of tests are publicly available through the open data platform (https://epistat.sciensano.be/covid/). However, not all the raw data used in this study are included in the open data. The datasets generated and analyzed during the current study are not publicly available due to request compliance with the European General Data Protection Regulation (GDPR) and privacy concerns but are available on reasonable request. A procedure to ensure fair data access has already been implemented through a structured data request form (https://epistat.sciensano.be/datarequest).

## List of abbreviations

NPI: non-pharmaceutical interventions
RI: real incidence
OI: observed incidence
PR: positivity ratio
SCC: suspected COVID-19 case
HRC: high risk contact
